# Effects of COVID-19 on Older Adults: Physical, Mental, Emotional, Social, and Financial Problems Seen and Unseen

**DOI:** 10.7759/cureus.29493

**Published:** 2022-09-23

**Authors:** Brianna Cocuzzo, Algevis Wrench, Chasity O’Malley

**Affiliations:** 1 Pediatrics, Maria Fareri Children’s Hospital, Valhalla, USA; 2 Microbiology, Nova Southeastern University, Fort Lauderdale, USA; 3 Medical Education, Nova Southeastern University Dr. Kiran C. Patel College of Allopathic Medicine, Fort Lauderdale, USA; 4 Medical Education, Wright State University Boonshoft School of Medicine, Dayton, USA

**Keywords:** older adults, isolation, mental health, covid-19, financial impact, support networks, covid-19 retro

## Abstract

Older adults are vulnerable to coronavirus disease 2019 (COVID-19) and efforts have been made to protect them. However, one protective mechanism, isolation of older adults, has resulted in unintended physical, mental, emotional, social, and financial consequences. We conducted a comprehensive literature review to understand the effects of COVID-19 and the new COVID-19 vaccine on older adults and the factors affecting vaccine acceptance. A review of the literature was conducted to understand the unique challenges COVID-19 creates for older adults. It was found that older adults are significantly impacted by the COVID-19 pandemic and resulting isolation. Physical health can be improved by increasing contact with healthcare providers and opportunities for physical activity. Mental and emotional health can be improved by addressing fear and uncertainty about the pandemic. Social health interventions should be targeted at ensuring older adults have contact with loved ones both in person and in the virtual format. To ameliorate financial concerns, interventions should be targeted at assistance with food and medications.

## Introduction and background

In December 2019, the reports of a novel virus causing symptoms ranging from mild respiratory infection to pneumonia, to severe acute respiratory distress emerged from Wuhan, China, and prompted global concern. Since that time, this novel virus, known as coronavirus disease 2019 (COVID-19) or, SARS-CoV-2, has spread across the world. The virus, which was declared a pandemic on March 11, 2020, has caused not only disease but also economic destruction [1-2]. There is clear evidence that the severity of the coronavirus disease varies across age, gender, and health status leaving older individuals, especially those with multiple comorbidities at a higher risk for severe complications and long-term health consequences including death [3-5]. The severity of illness is further increased in older adults as they have decreased immune function. While the risk of death in the general population infected with the coronavirus is low, it increases exponentially with age with the risk of death being as low as 0.1% in children and as high as 14.8% in older adults, representing an increase of more than 100-fold across the lifespan [6]. This indicates a need for stringent infection control measures for older adults [6]. As a result of this, older individuals are being urged to engage in social distancing, avoid non-essential travel, and isolate themselves in their homes to a greater degree than their younger counterparts. The Centers for Disease Control and Prevention (CDC) has made several strong recommendations regarding the measures older adults should take including stocking up on essential items such as non-perishable foods, personal hygiene products, and medications to last up to 90 days to avoid trips to the grocery store or pharmacy [7]. Similar recommendations exist for vulnerable older adults around the world. For example, in the United Kingdom, elderly individuals or those with pre-existing conditions received a "shielding letter" urging them to avoid contact with others including avoidance of grocery stores, avoidance of common areas such as the kitchen and shared bathrooms in the house, and refraining from sleeping with other members of the household in the same bed where possible [8]. Although these measures have reduced the transmission of COVID-19 to older adults, they also contribute to isolation and anxiety.

Nursing homes have taken steps to protect their residents. For example, the CDC has made several recommendations for nursing homes in the United States, including restricting all visits aside from end-of-life compassionate care, cessation of all activities requiring non-essential services, cancellation of large group activities including group meals in a communal dining hall, screening of all visitors of respiratory symptoms, and scheduling of visits, and limitations on the number of visitors per patient [9]. Nursing homes have also instituted policies that require regular screening of all patients and care providers for fever and respiratory symptoms to allow isolation of ill patients and removal of ill employees until they return to health [9]. Nursing homes are also being encouraged to provide paid sick leave so employees do not report to work sick due to fear of losing their job or losing pay [9]. Staff reporting to work ill poses an enormous risk to the patients. Although symptoms may be milder or more manageable for younger, healthier employees, the consequences for older adults with multiple comorbidities can be catastrophic [9]. Given that older adults are at higher risk of morbidity and mortality from COVID-19, they should not be exposed to infected staff members [9]. Additionally, patients in nursing homes are encouraged to delay non-essential medical appointments and/or to conduct visits using telehealth when possible [9].

Although in some areas, restrictions are beginning to lift, social isolation, social distancing, and additional safety regulations are expected to be needed, especially for older adults. This continued isolation is especially concerning given the fact that older adults are at increased risk for adverse effects of social isolation such as worsening physical conditions, neurocognitive dysfunction, depression, falls, and increasing frailty than their younger counterparts [1,10-12]. In the United Kingdom, concern has started to emerge regarding the potential that should isolation continue for an extended period, it could produce unintended harm for the elderly due to the increased risk of autoimmune, cardiovascular, neurocognitive, and mental health problems [13]. Following this investigation, a recent study in the United States elicited the same concerns [10]. Social distancing is, of course, effective to slow the spread of SARS-CoV-2 and shielding is especially important for those at the highest risk [10]. Making recommendations for the elderly is proving difficult; while the elderly do belong to the high-risk group, they are also more likely to suffer from increased morbidity and mortality because of withdrawal from social contact and mental stimulation. We must protect them from the coronavirus but also from these unintended consequences [13]. These risks will be especially pronounced in those older adults who are retired, live alone, or rely on adult care facilities, senior centers, or places of worship for their only social relationships. While social connectedness can be achieved through technologies such as the telephone, social media, and online chat platforms such as Zoom, Facetime, and Google hangouts, there are disparities both in access and ability to use these technologies among the elderly [14]. While this problem can be mitigated to some extent by ensuring frequent phone conversations with older friends and relatives who are not comfortable with using newer technologies to communicate, isolation produces tangible consequences for those adults who are the most isolated [10].

Methods

This review of the literature was conducted as part of a larger study designed to understand the unique challenges facing older adults because of the COVID-19 pandemic and to provide strategies to mitigate them. A database search was performed using Pubmed, Google Scholar, and the Nova Southeastern University Library Database for those articles related to the impacts on the physical, mental, emotional, social, and financial well-being of older adults. The search terms used were “COVID-19” “geriatric patients” “older adults” “adults over 65”, “impact of COVID-19 on older adults” and “social isolation.” The abstracts were screened to ensure they specifically studied adults over 65 and examined the unique hardships they face because of COVID-19.

Then, the full text of the articles was analyzed and focused on the stated difficulties faced by older adults and the impacts on their physical, mental, emotional, social, and financial well-being. The analysis was qualitative rather than quantitative as the articles focused primarily on the experiences, reports, and observations of older adults during the COVID-19 pandemic.

## Review

We discovered that older adults are impacted physically, mentally, emotionally, socially, and financially by COVID-19 and their anxiety surrounding the virus but perhaps more so by the effects of isolation due to restrictions to prevent the spread of the virus. Based on this, we make recommendations to balance the need to reduce the spread of COVID-19 and keep older adults safe from its effects and the needs to maintain their physical, mental, emotional, social, and financial well-being throughout the pandemic and beyond.

To best maintain the physical health of older adults, we recommend physical activity for older adults in a safe, isolated setting, even if the use of gyms or other public areas is not possible. We also recommend telemedicine visits to maintain the well-being of low acuity patients but the possibility for in-person visits, even for vulnerable older adults that require more acute care. To preserve the mental and emotional well-being of older adults, we recommend programs, virtually, or in person, to safeguard their mental health and address the issues of anxiety, depression, and thoughts of suicide. For example, older adults should be provided the opportunity to virtually participate in training and workshops on important wellness, mindfulness, and self-help skills such as meditation demonstrated to improve mental health. To protect the social well-being of older adults, we recommend reducing social isolation through human touch and activities to help older adults engage, at least virtually, with their friends and family. We strongly recommend human touch to improve social well-being. A recent study spoke to primary care physicians and patients, and physicians astutely noted that the benefit of human touch for elderly people is enormous. One doctor stated “Older people respond or seem to benefit from skin to skin. Just holding hands while you talk about how they’re feeling particularly” [14]. We need to make sure, even when families cannot be inside to hold older adults' hands, or touch their arms while they converse, that this human touch is available. That may require training staff to provide therapeutic, rather than procedural, touch to older adults, especially in long-term care settings as part of routine care [10]. To enhance the financial well-being of older adults, we recommend recognition of the enormous financial impact COVID-19 has on older adults. We must address the difficulties older adults face in accessing food and medications by making available, and increasing awareness of, programs for financial assistance for food and medications for older adults unduly affected by the pandemic. Furthermore, we should provide support for older adults to participate in these programs, particularly those with physical or cognitive limitations that make participation difficult.

Physical impact of isolation

The problem of poor physical health due to isolation is further exacerbated by the unique challenges that older adults face with access to medications and health care. For example, older adults with multiple comorbidities lack their normal access to primary care visits. In the United Kingdom, physicians, who work for the National Healthcare System, were advised that they may, and in fact, should suspend welfare checks for adults over the age of 75, through at least October 2020 at the start of the pandemic. In addition, physicians were informed that the government would make once-off adjustments to their payment should they earn less than their usual income due to the decrease in wellness checks while helping with the pandemic [15]. The CDC in the United States has also published several recommendations regarding the increased use of telemedicine in response to the COVID-19 pandemic [2]. They maintain that telemedicine is the safest way to provide patients with continuous care while reducing transmission [2]. Telemedicine is strongly recommended among low acuity patients to prevent overcrowding of hospitals and emergency rooms due to unnecessary hospital visitations, and human contact. While this change was effective in reducing overall mortality in patients with poor access to care and preventing the spread of disease, data from both China and Italy indicates that telehealth may not be sufficient or practical for the most vulnerable older adults who may be uncomfortable with technology or have complex conditions [2]. Telemedicine cannot replace a full physical exam and may be confusing and overwhelming for older patients leading to decreased quality of care. Therefore, the pandemic poses a risk to physical health even in those older adults who are not directly affected by COVID-19. Additionally, many older adults are experiencing a worsening of their physical health during the pandemic due to restrictions requiring them to stay at home and away from other people, limiting outdoor exercise, use of gyms, and curtailing attendance at therapies. The increasingly sedentary lifestyle is associated with cognitive decline, increased cardiovascular disease, poorer insulin sensitivity, and overall increased mortality [14]. In older adults, the physiologic changes associated with a decreased level of physical activity led to a lean muscle mass loss of as much as 4% over the course of just two weeks in older adults, this is particularly problematic as they have a reduced number of satellite cells and therefore, are less able to produce new myonuclei to regenerate muscles after atrophy or damage [16]. Therefore, the limitations on physical activity produced by the shutdown pose a major threat to the health of older adults. It’s critical to ensure that older adults can maintain some degree of physical activity [16].

Mental and emotional impact of isolation

This problem of worsening mental health in the elderly due to social isolation is of particular concern for those with prior histories of mental health problems [11]. Social network ties, social network structure, and participation in community events are demonstrated to help the mental health of older adults. Older adults who are socially isolated experience changes in their mood, cognition, and sensitivity to threats as well as disruptions in sleep, and older adults who feel alone report more depressive symptoms than their socially connected counterparts [17]. Reports are emerging from India of cases of older adults with prior history of mental health disorders presenting to the emergency room with severe exacerbations of symptoms specifically attributed to social isolation and decreased access to mental health services during the COVID-19 pandemic [11]. This overburdens psychiatrists, and already overwhelmed emergency departments increasing wait time, burn-out among staff, and exposure to COVID-19 [11]. In China, where the COVID-19 pandemic began, social isolation, distancing, and a complete lockdown began earliest, resulting in a wide breadth of research on the impact of social isolation related to the COVID-19 pandemic on society, including the elderly. According to research in China, 37.1% of seniors experienced symptoms of depression and anxiety during the COVID-19 pandemic with those most vulnerable to the effects of depression and anxiety being women, those who are widowed, or divorced, live alone, have worse physical health or have sleep problems [1]. Additionally, as expected, Chinese data verified that due to the increased severity of illness and mortality in older adults, the impact on both their physical and mental health was more obvious in older adults [1]. It’s imperative to develop appropriate, accessible interventions to ensure the mental health of the elderly is preserved throughout the pandemic [1,3]. Data from China can provide a useful perspective on the potential needs of older adults and potential interventions to address them. The importance of mitigating the impact of the COVID-19 pandemic on the mental health of older adults is underscored by the research which emerged from Hong Kong demonstrating increased rates of suicide among older adults associated with the 2002 SARS outbreak in Hong Kong [16]. Due to the severity of SARS in older adults and the widespread public fear surrounding the SARS outbreak, the suicide rate in Hong Kong among older adults significantly increased in the peri-SARS period peaking in April 2003, directly coinciding with the peak of the SARS outbreak [16]. The spike in older adult suicides represented an alarming increase of 31.7% higher than the rate in 2002 when the outbreak was first declared [16]. Perhaps most alarmingly, rates of suicides among older adults persisted beyond the end of the SARS outbreak and persisted even beyond the time necessary for economic recovery [16]. This demonstrates the severe impact that fear, isolation, and uncertainty surrounding the pandemic. Interventions should be specifically targeted at improving access to mental healthcare, especially emergent intervention for patients considering suicide.

Social impacts of isolation

Older adults are more vulnerable to the physical effects of COVID-19 and significantly more likely to succumb to COVID-19, especially if they have comorbid conditions. There is a strong correlation between the estimate of personal risk and willingness to return to normal life as restrictions lift. [17]. Additionally, older adults tend to be less comfortable with virtual alternatives to face-to-face communication than younger people. Therefore, they don’t communicate with each other or with their younger family members virtually [2]. As begin to reopen and the option for normalcy slowly emerges, older adults may remain isolated. Social isolation causes a variety of problems for older adults including depression, anxiety, cognitive dysfunction, heart disease, and mortality [18]. According to the former surgeon general, Vivek Murthy, loneliness is a major concern for older adults [19]. He states, “the most common pathology I saw was not heart disease or diabetes; it was loneliness” [19]. He further concluded that loneliness is associated with a huge reduction in life span which is estimated to be greater than the reduction in lifespan associated with obesity and equal to smoking 15 cigarettes per day [19]. Consequently, it is critical to take advantage of the willingness of some older adults to learn how to utilize these communication technologies to include them in virtual communication activities [2]. Additionally, for those older adults least able to engage with new technologies, such as those living in long-term care or assisted living facilities, it is important to provide human touch and other forms of social contact, with staff filling in when family cannot be present.

Financial impact of isolation

In addition to the impact on the physical and mental health of older adults, coronavirus produces a variety of financial problems for older adults who are already at risk for financial hardship in times of recession [4]. Older adults are more vulnerable to the effects of economic downturns and recessions, especially those which are rapid and unexpected, than their younger counterparts due to the decreased time until retirement to recover financially. As seen in the 2008 economic recession, older adults experience significant declines in net worth during economic downturns [4]. Older adults in the 65-74 year age bracket and those who are still working are hardest hit by the economic recession. This may be because older adults who are not yet retired are less likely to be stable on social security and those who are in the younger age bracket are less likely to have finished paying off their mortgage [4]. The impact of economic recessions is most severe in the individuals in the lowest quintile of wealth [4]. This trend is expected to continue in the COVID-19 pandemic as the poorest older adults will be least able to manage should they be furloughed or afraid to work due to illness or fear of infection [4]. Alternatively, those lower-income older adults may risk their health to continue working despite their increased vulnerability. For many lower-income older adults, economic instability is a food security issue. The CDC recommends stocking up on three months’ worth of food, but this is impossible for many older adults who already have difficulty affording or accessing adequate healthy foods that meet their daily needs. In addition to the inability to stock up on food and supplies due to financial constraints, limitations on the amount of food purchased using Supplemental Nutrition Assistance Program (SNAP) benefits, or failure of older adults to use SNAP benefits due to embarrassment, lack of awareness of their eligibility, and difficulty navigating the application process contribute to food insecurity in older adults [5]. Even if homes with older adults do apply for SNAP benefits, many of them still receive only the lowest level of benefit, $16 a month, hardly enough to stock up [5]. Services such as food banks and community meals at senior centers have been suspended or older adults are afraid to utilize them. Many older adults who require specialized diets for chronic conditions, such as diabetes and heart disease, cannot get nutritious food that meets their needs, leading to unnecessary hospital visits and increased healthcare utilization, especially given the limitations on doctor’s visits for chronic conditions [4-5]. It’s prudent to prevent these outcomes due to the high burden COVID-19 places on the healthcare system.

Further complicating the issue, the presence of chronic medical conditions is a major economic concern for older adults. In fact, medical bills are the single largest driver of debt in households with older adults in the United States [5]. Medications comprise approximately 16% of healthcare costs in the United States and the average number of drugs prescribed for patients 80 and older is a staggering 22 medications including those for diabetes, hypertension, heart disease, and cancer [20]! Adults 65-79 cover approximately 56% of these costs out of pocket while those 80 and older pay a whopping 67% [5]. Sadly, 22% of older adults report failing to fill one or more prescriptions because of cost, and 23% report intentionally skipping doses of required medications to stretch them [20]. The economic impact of COVID-19 poses an enormous risk to older adults who can’t comply with CDC recommendations to stockpile medications and in many cases, are forced to go without. We must develop programs and increase awareness of programs that allow older adults to access food and medications at a discounted price or provide temporary assistance to those who have reduced income due to COVID-19.

What does the vaccine mean for older adults?

The long-awaited COVID-19 vaccine is now widely available. This provides a glimmer of hope for older adults. When the population is vaccinated and herd immunity is reached, the economy can recover. However, even the idea of lifting restrictions once herd immunity is established is fraught with challenges. For example, the establishment of herd immunity is inherently based on long-term immunity, despite emerging evidence of reinfection [7]. Additionally, before we can achieve herd immunity, we run the risk of overwhelming the healthcare system and leaving people without access to healthcare. In addition, those most vulnerable to the effects of the disease, including older adults may experience significant morbidity and mortality before herd immunity is achieved [7]. Of course, to even reach the herd immunity required to keep older adults safe, a vaccine that is safe, effective, tested enough to be widely accepted, and produces immunity that lasts. Furthermore, there is evidence emerging that vaccinated people are not protected from nasal infection and therefore, are still capable of asymptomatically spreading the virus [21]. Unfortunately, barriers remain to providing a safe re-entry to society for older adults.

While the COVID-19 vaccine may provide some protection, it has several limitations. First, people who are vaccinated may be colonized with COVID-19. While they are not generally as symptomatic, there are reports of “breakthrough” cases with sick, and contagious, vaccinated patients [21]. The purpose of any viral vaccine, including an mRNA vaccine like those used for COVID-19 is to generate a cellular immune response that effectively prevents systemic replication and symptoms of respiratory infection. These vaccines, however, provide limited mucosal protection [21]. To prevent replication within the mucosa, we would really need a mucosal vaccine, such as the oral polio vaccine or intranasal influenza vaccine, options that do not currently exist for the COVID-19 vaccine [21]. It is not surprising, therefore, that elevated nasal titers continued to be reported in people without symptoms and they continued to spread the virus via respiratory droplets [21]. This leaves people who cannot or do not wish to receive the vaccine or those with a waning response to the vaccine vulnerable to COVID-19 [21]. 

Considering this, is the COVID-19 vaccine the silver bullet for older adults we once thought it was? Unfortunately, many of those individuals who have a suboptimal immune response to the COVID-19 vaccine and therefore, remain vulnerable are older adults [22]. Research indicates that while achieving immunological protection which can eliminate or severely limit the capacity of the virus to disseminate to host cells may be possible in younger people, this may be a challenge in older adults. Historically, the seasonal influenza vaccine is less efficacious in older adults [22]. As people age, our immune system weakens via a variety of mechanisms. Most importantly for vaccine development, the ability to develop adaptive immune responses is compromised in older adults as antigen-presenting cells do not function as well as they previously did, meaning they cannot generate a robust immune response. At the present time, there is no guarantee that a vaccine developed conventionally based on young people’s immune response will be effective in older adults [22]. Even among younger people, with a more pronounced immune response, who are vaccinated, there is concern that immunity is waning and the CDC is recommending booster vaccines [23]. This begs the question, are vulnerable older adults even well protected?

Then, of course, there is the issue of herd immunity. We cannot achieve herd immunity without vaccinating most of the population. Even as the vaccine becomes widely available, many Americans do not want to be vaccinated. Many Americans are distrustful of the COVID-19 vaccine, particularly among African Americans and other minority groups who have a distrust of the healthcare system due to differences in health outcomes and concerns over prior victimization with clinical trials [24]. Even among healthcare providers, there is slow uptake of the vaccine. Many of these individuals expressed concern about the safety of the vaccine, given how rapidly it was developed. Although the self-perceived risk of severe COVID-19 infection was a major predictive factor for vaccine acceptance, age is not [25]. Furthermore, clinicians who are not caring for COVID-19-positive patients trust the COVID-19 vaccine even less than the general population. A survey given to both healthcare workers and civilians demonstrated that the primary reason for refusal of the COVID-19 vaccine or vaccine hesitancy was concern over quality control and side effects [25]. Many participants also cited concerns that politics are taking on greater importance for vaccine developers and other stakeholders than the science behind the vaccine and believe there was a rush to finish the vaccine before the US general election of 2020 [26]. An additional study indicated that nurses are generally slower to accept vaccines than doctors and that many healthcare workers are willing to get the vaccine but plan to “delay” their vaccination as they feel the development was too rushed and are not confident; they understand the long-term effects of the vaccine. Of note, these responses came from healthcare workers who had very high confidence in the safety and efficacy of vaccines in general and understood the importance of routine vaccinations for themselves and others: their concerns were unique to the novel coronavirus vaccine [27]. 

Therefore, the vaccine may not be the panacea we are hoping for and the need for social distancing and isolation of older adults will continue, at least for now. Consequently, it is incredibly important to exercise these recommendations to protect and preserve the lives and livelihood of our most vulnerable not just by protecting them from COVID-19 but by ensuring their well-being in all aspects of life.

Due to concerns particularly about the spread of COVID-19 in the setting of long-term care facilities due to both difficulty with social distancing and decreased immunity in older adults, there has been a push to vaccinate employees of care facilities to keep institutionalized older adults safe. However, this too is fraught with difficulty. Hesitancy due to concerns about the safety of the vaccine is most pronounced in nursing homes. Healthcare workers, who were offered the vaccine first, are afraid to accept it [28]. Currently, nursing home employees are less likely than their counterparts working in other areas of healthcare to accept the seasonal influenza vaccine, with an acceptance rate of only 69.3%, lower than those working in primary care settings and those working in hospitals [29]. The same may be true for the coronavirus vaccine. If this is the case, older adults in the long-term care setting face insurmountable challenges, and true protection from COVID-19 is likely not in their immediate future.

Implications and future directions

So, is there a better option for older adults? Is quarantining them worth it? While most countries have put strict lockdowns and social distancing measures into effect, Sweden has remained mostly open and has opted to allow individual citizens to make their own decisions regarding staying at home, exercising social distancing, and limiting contact with others, however, they did recommend but did not mandate, a voluntary shelter in place for adults over the age of 70 [28]. When researchers in Sweden spoke to older adults, the results were surprising. Generally, subjective health declines with age, however, most people in the survey rated their well-being as “just as high” or “higher” than it was in previous years [28]. Those who worried more about health and financial consequences tended to report lower well-being. For older adults, being up and about and engaging in normal daily activities is important to maintain a sense of well-being [28]. If we examine the situation critically, the vaccine likely isn’t sufficient to protect older adults as there is still the risk of asymptomatic carriage, people are slow to accept the vaccine, and older adults may not have the immune response needed for the vaccine to be effective.

## Conclusions

This review of the literature was written to help provide an understanding of the unique challenges facing older adults because of the COVID-19 pandemic, as well as to provide strategies to mitigate them. Overall, the COVID-19 pandemic, and the isolation which resulted from social distancing, impacted older adults in ways beyond social aspects. This review highlighted the social aspects of isolation but also shed some light on the physical, emotional, mental, and financial aspects of life which were affected by the isolation induced by the pandemic. Support for older adults to participate in financial assistance programs and in social activities can help to alleviate some of the implications of isolation. With vaccines becoming more a part of life, individuals are able to resume visits with their loved ones and to return to physician offices in person for visits more regularly. However, while the vaccine may provide a glimmer of hope, the truth is, we are far from being able to guarantee older adults their pre-COVID quality of life. We must foster social connections and reduce anxiety among older adults to provide hope, regardless of the trajectory of the pandemic. The literature provides wonderful suggestions to enhance the well-being of older adults at the tail-end of the pandemic and beyond!
